# Precision medicine and personalized nursing in cardiovascular disease: clinical applications and frontier developments

**DOI:** 10.3389/fcvm.2026.1616059

**Published:** 2026-02-19

**Authors:** Xiaolan He, Guiying You

**Affiliations:** Department of Cardiology, West China Hospital, Sichuan University, Chengdu, Sichuan, China

**Keywords:** artificial intelligence, cardiovascular disease, omics technology, personalized nursing, precision medicine

## Abstract

Cardiovascular diseases (CVDs) continue to be the leading cause of mortality worldwide, and the available treatments are not sufficiently effective. The integration of precision medicine with personalized nursing offers a promising approach for early prevention, accurate diagnosis, treatment optimization, and prognosis assessment in CVD management. This review systematically analyzes recent advancements in multi-omics analysis and artificial intelligence (AI) technologies within the cardiovascular field, focusing on how precision medicine can improve the diagnostic and therapeutic accuracy as well as care efficiency, thereby improving clinical outcomes. For personalized nursing, we propose a novel implementation framework that incorporates physiological, psychological, social, and cultural dimensions, creating an integrated patient-centered management model based on comprehensive profiling. The synergistic integration of precision medicine and personalized nursing can yield a collaborative system that allows precise identification, individualized intervention, and comprehensive management, reflecting a paradigm shift toward patient-centered healthcare. In addition, the present analysis reveals existing challenges—including data privacy, ethical considerations, and cost-effectiveness—underscoring the urgent need for interdisciplinary collaboration and technological innovation to overcome these barriers. Therefore, the insights from this study are expected to guide the formulation of future research aimed at developing optimal clinical interventions to improve the prognosis and quality of life for patients with CVD.

## 1 Introduction

Cardiovascular diseases (CVDs) represent a group of disorders that significantly impact the heart and blood vessels, such as coronary artery disease (CAD), stroke, peripheral arterial disease, and congenital heart defects (1, 2). According to data from the World Health Organization (WHO) in 2019, CVDs contribute to approximately 17.9 million deaths annually, representing 32% of global mortality. The high prevalence of CVDs not only imposes a substantial economic burden on families and society, but also poses a significant challenge to the utilization of healthcare resources. Traditionally, the management of CVDs is based on population-based guidelines and standardized therapeutic protocols (3–5). Although they are effective in reducing disease incidence, the high heterogeneity among individuals in terms of genetics, lifestyle choices, and environmental factors necessitates the search for establishment of targeted treatment strategies. Therefore, precision medicine and personalized nursing have emerged as pivotal directions within cardiovascular nursing.

Precision medicine allows individualized treatment strategies by integrating patient-specific clinical data, lifestyle factors, genetic characteristics, and biomarkers (6, 7). The rapid advancements in molecular biology during the 1950s has revealed deeper understanding of the relationship between genes and diseases, laying a theoretical foundation for precision medicine. In 2011, the U.S. National Research Council formally introduced this concept in its report “Toward Precision Medicine”, proposing a novel classification system based on molecular biology that incorporates information technology and multi-source data to redefine diseases at the molecular level (8).This marked a major shift from population-based to individualized treatment, providing a promising approach for the treatment of complex diseases such as CVDs and advancing personalized nursing practices.

The on-going advancements in the field of medical diagnostics and the deep integration of AI into global healthcare systems, coupled with the application of next-generation technologies have revolutionized the clinical management of diseases. In medical imaging (9, 10), deep learning has improved ultrasound image segmentation, significantly optimizing diagnostic accuracy and efficiency (11). In biomedical signal processing (12, 13), AI technologies have not only optimized data acquisition workflows but also improved model generalization capabilities while substantially reducing computational costs (14). Meanwhile, in interdisciplinary fields such as earth sciences (15, 16), AI-powered deep learning models have enabled intelligent identification and classification of microcrystals, improving Scanning Electron Microscopy (SEM) image processing efficiency through high-performance segmentation and precision analysis (17). Regarding personalized nursing for CVD patients, emerging technologies, including multi-omics, artificial intelligence, and big data analytics, are reshaping the development trajectory of this discipline. In recent years, disease molecular subtyping has evolved from relying on a single or few biomarkers to the use of a more precise classification based on comprehensive omics feature profiling and integrative multi-omics analysis. This new technology allows a deeper exploration of patients' genetic information, metabolic profiles, and protein expression levels, providing a robust foundation for disease diagnosis, treatment optimization, and prevention (18–20). Moreover, the integration of AI and big data analytics has enabled researchers to develop models for processing multidimensional data to identity novel diagnostic and prognostic biomarkers for CVDs (21, 22).

Personalized nursing, as a patient-centered nursing model, can tailor interventions based on individual differences, aiming to enhance care precision, optimize clinical outcomes, and reduce hospital readmission rates (23, 24). Numerous investigations have shown that personalized nursing improves health outcomes, increases patient adherence to treatment, and enhances nursing quality (25–27). Personalized nursing achieves precision treatment through multidimensional individualized interventions as follows: (1) Precision risk assessment: Integrating genomic data, biomarkers, and clinical characteristics to construct predictive models using machine learning techniques for identifying high-risk individuals. For example, a Polygenic Risk Score (PRS) has been developed for predicting the risk of early-onset CAD (28, 29); (2) Optimization of treatment plans: Developing individualized therapeutic strategies based on pharmacogenomics (e.g., VKORC1-guided warfarin dosing) and pathological classifications [e.g., employing SGLT2 inhibitors specifically for heart failure (HF) with preserved ejection fraction - HFpEF], which minimizes adverse drug reactions (30, 31); (3) Dynamic health management: Utilizing wearable devices (e.g., ECG patches) for real-time monitoring and digital therapeutics (e.g., AI health assistants) facilitates closed-loop management, with remote monitoring reducing HF readmission rates by up to 30% (32, 33). These approaches creates a novel “Predictive, Preventive, Personalized, and Participatory” (P4 medicine) paradigm, with superior outcomes to traditional empirical medicine (34, 35). Although significant advancements have been made in the field of CVD research, the application of precision medicine in clinical personalized nursing practice still faces multiple challenges. For instance, most studies have investigated precision medicine and personalized nursing in isolation, paying little attention to their synergistic effects. Moreover, with the rapid development of AI and multi-omics technologies, systematic evaluation of their value in enhancing the precision diagnosis and individualized treatment of CVD is lacking. To overcome these research bottlenecks, this review aims to: (1) intergrate multi-omics technology systems (including genomics, transcriptomics, metabolomics, proteomics, and network medicine) with AI algorithms to comprehensively analyze their synergistic mechanisms in personalized cardiovascular care; (2) develop a multidimensional nursing model that incorporates physiological indicators, psychological status, social support, and cultural background; (3) critically examine the core methodological issues such as data bias, cost-effectiveness, and healthcare equity to identify avenues for deeper integration of precision medicine and nursing practice; and (4) provide actionable clinical translation strategies for interdisciplinary teams, that will improve patient outcomes.

To achieve these objectives, we follow a progressive framework of “multidimensional analysis—technological integration—clinical implementation”. First, a how multi-omics technologies reveal the molecular heterogeneity of CVD is described to provide a theoretical foundation that will promote precision subtyping. Next, AI and big data analytics integrating multimodal data through algorithmic processing are explored, allowing the translation of molecular signatures into clinically actionable information. Finally, personalized nursing plans that utilize these discoveries to individual patients, are discussed, thereby completing the “mechanism-to-intervention” cycle. In this workflow, multi-omics enables “discovery”, AI facilitates “translation” and nursing practice accomplishes “application”—collectively driving the transformation of cardiovascular medicine from traditional experience-based paradigms to data-driven precision models.

## 2 Application of precision medicine in CVDs

In 2022, the Association of Cardiovascular Nursing & Allied Professions (ACNAP) of the European Society of Cardiology issued a statement on comprehensive cardiovascular care (36), emphasizing patient-centered cardiovascular nursing that integrates multidisciplinary resources and adopts systematic approaches to ensure continuity of care, and improve patient outcomes and quality of life. The statement highlighted the critical role of emerging technologies in integrated care. For instance, the application of omics technologies enhances diagnosis and personalized treatment, improves early warning systems, intervention capabilities, prognosis evaluation, and long-term management of CVDs. Additionally, AI has been employed to support self-care among patients with chronic heart failure (CHF), through programs, such as virtual “family doctors” to educate and empower patients (37, 38). In the era of big data, medical decision-making has traditionally relied on population-based research and clinical experience, often overlooking individual differences, which potentially affect diagnostic and therapeutic inaccuracies. Precision medicine addresses this limitation by integrating diverse data sources such as electronic health records (EHR), medical imaging, and genomic sequencing, enabling a more comprehensive understanding of patient conditions and the formulation of tailored medical strategies.

In this chapter, we adopt a dual framework integrating technology-driven and clinical application-oriented approaches, leveraging a progressive structure that transitions from basic research to clinical implementation, and from molecular mechanisms to intervention strategies. With reference to the precision medicine research paradigm—“discovery-validation-translation”, we systematically describe omics technologies (genomics → transcriptomics → metabolomics → proteomics → network medicine) and AI technologies (diagnosis → treatment management → workflow optimization → prevention), formulating a comprehensive pipeline that spans from technological foundations to clinical applications.

### 2.1 Omics technologies in cardiovascular precision medicine

Recent advancements in omics technologies and multi-omics data has led to the discovery of CVDs biomarkers, making precision treatment and personalized nursing feasible (19, 39, 40). Genomic data may carry genetic risks associated with CVDs through sequence analysis; transcriptomics and proteomics provide molecular insights into disease mechanisms; metabolomics elucidates metabolic dysregulation; and network medicine fosters a systematic understanding of disease pathways. Although each omics layer offers unique advantages and targeting specific disease features, single-omics data alone cannot reflect the entire pathological process. Therefore, multi-omics integration enables the identification of pathological mechanisms, pathways, biomarkers, and other relationships by aggregating datasets from different omics layers (19, 41). This provides a more comprehensive and accurate molecular profile of CVDs, thereby facilitating early diagnosis, precision treatment, and personalized nursing. In this section, we will systematically review the role of multi-omics technologies in cardiovascular research and their potential to improve personalized nursing (see Table 1).

**Table 1:** Application of multi-omics technology in precision medicine.

Disease type	Application case	Key findings	Clinical significance	Author information
Genomics				
PAH	Rare variant analysis in 4,241 PAH patients identified multiple rare genetic variants associated with PAH.	Identified PAH-related rare genes, providing new insights into disease mechanisms.	Facilitates early diagnosis and personalized treatment of PAH.	Zhu et al. (46)
DCM	Investigation of the genetic mechanisms of DCM, analyzing 9,365 cases and 946,368 controls.	Identified 70 DCM-associated genomic loci mapped to 63 prioritized genes.	Elucidates the genetic architecture of DCM and aids in identifying novel therapeutic targets.	Jurgens et al. (47)
CAD	Analysis of atherosclerotic lesions revealed CAD-related variants enriched in specific cell types.	Identified CAD-related regulatory elements.	Helps pinpoint pathogenic mechanisms of CAD.	Örd et al. (49)
CVD	Bidirectional Mendelian randomization study using cognitive performance data from large-scale GWAS datasets.	Cognitive impairment may increase the risk of atrial fibrillation, HF, and other CVDs.	Provides genetic evidence linking CVD and cognitive impairment, offering novel perspectives for CVD prevention.	Lincheng Duan et al. (50)
CAD	Analysis of genotype data from 11 GWAS cohorts, including 37,997 CAD cases and 42,854 controls.	Identified 114 CAD-associated genes, including 18 novel genes.	Provides new insights into CAD pathogenesis and potential therapeutic targets.	Li et al. (51)
CVD	GWAS and PRS analysis.	Identified pathogenic genes and genetic variants associated with various CVDs.	Assists in CVD diagnosis, risk prediction, treatment guidance, and cascade screening, supporting personalized medicine.	Michael P. Gray et al. (52)
Transcriptomics				
CAD	Perturb-seq, CRISPRi, and scRNA-seq	Identified 43 CAD GWAS signals converging on the CCM signaling pathway and a novel gene, TLNRD1, interacting with CCM2.	Reveals new mechanisms of CAD and provides potential therapeutic targets.	Schnitzler et al. (54)
Atherosclerosis	scRNA-seq and bulk RNA sequencing of human atherosclerotic plaques from the GEO database.	Identified 7 C1Q-related differentially expressed genes and revealed SPI1 as a key transcription factor regulating these genes.	Provides new biomarkers and therapeutic targets for atherosclerosis diagnosis and prognosis.	Cui H-K et al. (55)
Cardiomyopathy (DCM, HCM)	RNA-seq analysis of heart tissue identified regulatory networks in DCM and HCM.	Identified transcriptional markers specific to DCM and HCM.	Facilitates disease subtyping and personalized treatment.	Liu et al. (56)
DCM	scRNA-seq and bulk RNA sequencing of cardiac tissue.	Identified transcriptional changes in eight cardiac cell types, with significant alterations in fibroblasts.	Provides potential targets for DCM diagnosis and treatment, facilitating clinical research and therapeutic advancements.	Adam Russell-Hallinan et al. (57)
Cardiomyopathy (DCM, HCM)	scRNA-seq analysis of left ventricular tissue revealed cellular heterogeneity in DCM and HCM patients.	Discovered key signaling pathways and cellular heterogeneity.	Promotes personalized and cell-targeted therapies.	Chaffin et al. (58)
Metabolomics				
ACS	Integrated metabolomics and gut microbiome analysis of 199 ACS patients.	Identified individualized metabolic abnormalities.	Promotes precision treatment based on metabolic features.	Talmor-Barkan et al. (61)
HF Risk Prediction	Long-term follow-up study using 1H-NMR spectroscopy to measure serum metabolites in the UK Biobank cohort.	Multiple metabolites were associated with disease onset, and models incorporating metabolomic data improved prediction performance.	Enhances HF risk prediction; metabolomics-based models may serve as clinical alternatives.	Rafael R. Oexner et al. (62)
CAD	Nested case-control study using the BIPass cohort with untargeted metabolomic analysis via LC-MS.	Identified 23 shared differential metabolites associated with cardiovascular events, mainly medium- and long-chain acylcarnitines.	Supports precise cardiovascular risk assessment and the development of a two-step metabolic prognosis strategy for clinical guidance.	Jiali Lv et al. (63)
LVDD in Type 2 Diabetes	Collected clinical data and echocardiographic measurements from 855 hospitalized type 2 diabetes patients, calculating NLR and assessing associations.	LVDD patients had higher NLR levels; NLR was positively associated with LVDD prevalence, though some associations diminished after adjustments.	NLR may serve as a potential marker for identifying LVDD in type 2 diabetes, helping prevent HF.	Xueyang Yang et al. (68)
STEMI	Glutathione infusion in STEMI patients reduced inflammation and protected cardiomyocytes.	Early and prolonged glutathione infusion improves post-PCI cardiac remodeling.	Enhances myocardial protection post-PCI.	Gaetano Tanzilli et al. (69)
Proteomics				
MR and Plasma Proteomics	Plasma protein measurement using Olink proximity extension assay; cohort analysis and MR analysis.	Identified 45 proteins associated with MI risk; MR analysis revealed potential causal relationships for 7 proteins, some of which showed opposite directions in cohort analysis.	Provides new insights into MI pathogenesis, aids in identifying potential therapeutic targets, and highlights the importance of including both sexes in clinical and preclinical studies.	Olga E. Titova et al. (71)
Acute MI with Malignant Ventricular Arrhythmias (AMIMVA)	Data-independent acquisition proteomics, principal component analysis, KEGG analysis, GO analysis.	Identified two proteins (vWF and FGG) with significant differences across all three comparisons and nine proteins significantly different between AMIMVA/control and AMIMVA/AMI groups.	Provides new insights into the diagnosis and pathogenesis of AMIMVA.	Jian-Liang Zhang et al. (72)
HF	Phosphoproteomics analysis in HF patients.	Identified phosphorylation site alterations in HF, revealing abnormalities in pathways such as intercellular adhesion.	Offers new molecular insights into the pathophysiology of HF.	Reitz et al. (73)
ASCVD	Plasma protein levels measured using the SomaScan v4 platform (∼5,000 proteins).	A risk score based on 70 proteins was significantly associated with ASCVD events.	Provides new predictive markers for ASCVD risk assessment, aids in clinical decision-making, and helps identify high-risk individuals more accurately.	Helgason H et al. (76)
ASCVD	Proteomics analysis: mass spectrometry (discovery phase), targeted analysis using Olink, SomaScan, and other technologies.	Identified proteins such as NT-proBNP, KIM-1, and lipids such as ceramides associated with ASCVD risk.	Enhances precise ASCVD risk assessment and supports personalized prevention and treatment strategies.	Nick S. Nurmohamed et al. (78)
Network medicine				
Cardiomyopathy	Patient-specific PPI networks integrating genomics and transcriptomics data predicted individualized disease mechanisms.	Identified disease network modules specific to patients.	Promotes personalized precision treatment.	Maron et al. (81)
MI	Tissue-specific network analysis revealed metabolic pathways post-MI.	Identified tissue-specific metabolic changes.	Supports precision treatment strategies for different tissues.	Arif et al. (82)
Multi-omics Integration	Integrated genomics, transcriptomics, proteomics, and metabolomics data to construct a PPI network and perform KEGG/GO enrichment analysis.	Identified 40 significant MI-associated metabolic pathways, such as bacterial response and HIF-1 signaling.	Provides insights into MI pathogenesis, aiding in the development of effective diagnostic and therapeutic strategies.	Xiaolan Liu et al. (83)
Myocarditis	Multi-omics analysis, PPI network construction, and KEGG/GO analysis.	Identified reproducible biomarkers across genomic and proteomic datasets, primarily related to inflammation.	Enhances understanding of myocarditis pathogenesis, aiding biomarker discovery and clinical applications.	Wang et al. (84)
Multi-omics Network Integration	Utilized the Mergeomics 2.0 platform to integrate multi-omics data, identifying potential biomarkers and key regulators of CVDs.	Identified novel biomarkers and key regulatory factors for CVDs.	Provides new tools and methodologies for precision cardiovascular medicine by integrating multi-omics data.	Ding J et al. (85)

#### 2.1.1 Genomics

Over the past two decades, advancements in genotyping technologies, reduced genome sequencing costs, and the integration of digital technologies in healthcare have generated the third revolution in medicine. Using this approach, researchers can identify informative biomarkers (21, 42). Single-cell whole-genome sequencing, deep whole-genome sequencing, and high-throughput genotyping technologies have enabled researchers to uncover genetic mechanisms of CVDs, facilitating personalized nursing (43, 44). For example, pulmonary arterial hypertension (PAH) is a fatal vascular disease with complex etiology, partially influenced by genetic factors, yet the fully landscape of its genetic mechanisms remain unclear (45). In 2021, Zhu et al. conducted a rare variant analysis of 4,241 PAH patients and identified seven genes significantly associated with idiopathic PAH, offering novel insights into PAH pathogenesis and potential therapeutic targets (46). Jurgens et al. performed a genome-wide association study (GWAS) on dilated cardiomyopathy (DCM) using 9,365 cases and 946,368 controls, identifying 70 DCM-associated genomic loci and mapping them to 63 priority genes. They employed PRS and whole-genome sequencing (WGS) to investigate the impact of rare variants, uncovering the critical roles of BAG3 and TTN genes (47). Sonia Shah et al. (48) identified 12 independent variants from 11 genomic loci that were significantly associated with HF (HF) via a GWAS and Mendelian randomization analysis, with 10 loci being previously unreported. Tiit Örd et al. (49) conducted single-nucleus chromatin accessibility analysis on human atherosclerotic lesions, which identified over 30 potential causal single-nucleotide polymorphisms (SNPs) and target genes for CAD, some of which were linked to smooth muscle cell phenotypes. Using publicly available GWAS data, Lincheng Duan et al. identified that lower cognitive performance increased the risk of hypertension, atrial fibrillation, and HF (50). Numerous studies (51–53) have shown that genomics can identify pathogenic variants and improve risk stratification, thereby advance the implementation of precision medicine, risk assessment, and personalized treatment.

#### 2.1.2 Transcriptomics

Transcriptomics research can reveal important insights into the molecular pathways underlying CVDs. Schnitzler et al. identified several GWAS variants linked to gene functions, among which were 43 CAD-associated GWAS signals converging on the cerebral cavernous malformation (CCM) signaling pathway, suggesting that CCM2 and TLNRD1 are involved in endothelial cell anti-atherosclerotic processes (54). By integrating single-cell and bulk transcriptome analyses, C1QA and C1QC were identified as key diagnostic genes for PAH, with SPI1 potentially serving as a critical transcription factor (55). Chia-Feng Liu et al. (56) conducted whole-transcriptome analysis of left ventricular tissues obtained from HF patients with different cardiomyopathies. They identified that the Notch and SEMA4C pathways were enriched in hypertrophic cardiomyopathy (HCM), and SEMA4C was a potential HF biomarker. Adam Russell-Hallinan et al. (57) analyzed DCM cardiac tissues using single-cell RNA sequencing (scRNA-seq) and bulk RNA sequencing, and observed significant transcriptomic changes in cardiac fibroblasts. Through *in vitro* validation tests, they uncovered the AEBP1's potential to activate fibroblasts, uncovering novel therapeutic targets for DCM. Chaffin et al. (58) performed single-nucleus RNA sequencing (snRNA-seq) on DCM, HCM, and non-failing heart samples, which led to the identification of transcriptional alterations and cellular sub-population changes in disease states, particularly fibroblast activation characteristics, which expanded the current understanding of pathomechanisms. However, challenges remain, such as variability in transcriptomic profiles across patient cohorts and the need for standardized data analysis and interpretation methods.

#### 2.1.3 Metabolomics

Biomarkers allow disease detection, assessment, and cardiovascular risk management. Metabolomics has become a powerful tool for identifying metabolic biomarkers (59). With significant technological advancements, including mass spectrometry, chromatography, and nuclear magnetic resonance (NMR) spectroscopy, the efficiency and reliability of metabolic analysis have been improved significantly (60). Multi-omics analysis of patients with acute coronary syndrome (ACS) revealed that the BMI prediction model based on serum metabolomics showed a higher predicted BMI than the actual value, and this difference was associated with the severity of type 2 diabetes (T2DM) and CAD (61). By analyzing data from the UK Biobank cohort, Oexner team (62) discovered that several metabolites in serum metabolomics were significantly associated with the onset of HF, which improved risk prediction models and risk stratification. Another study conducted non-targeted metabolomics analysis to explore cardiovascular event-related metabolic biomarkers in patients with CAD (63). They found that medium- and long-chain acyl-carnitines were associated with composite cardiovascular event outcomes, and glycerophospholipids were specific metabolites for HF progression, which may potentially enhance CAD risk prediction. Numerous studies have found that various metabolic-related indicators may influence heart function (64–66). For instance, the neutrophil-to-lymphocyte ratio (NLR) can reflect the balance of cellular events during heart repair, and lower NLR values reflect improved left ventricular remodeling and systolic function (67). Yang et al. (68) found that NLR predict a higher risk of left ventricular diastolic dysfunction (LVDD) in T2DM patients and may serve as a potential biomarker for identifying LVDD, thus, promoting risk stratification and early detection. These studies indicate that metabolomics indicators can be leveraged to evaluate cardiovascular disease severity and prognosis (69). However, the stability and reproducibility of metabolomics biomarkers in different populations, as well as interactions between metabolites and other biological factors need to be further clarified.

#### 2.1.4 Proteomics

Proteins, as products of gene expression, participate in diverse biochemical activities and serve as intermediate phenotypes for diseases, drug development targets, and important biomarkers for prognosis assessment and treatment stratification (70). In a study by Titova et al. (71) involving cohort studies and Mendelian randomization (MR) analyses, 45 proteins associated with myocardial infarction (MI) risk were identified, among which 13 exhibited sex-specific associations, which were predominantly more significant in females. Further MR analysis showed that seven proteins were causally associated with MI. Zhang et al. (72), utilizing data-independent acquisition (DIA) proteomics, analyzed 190 blood samples and found that patients with acute myocardial infarction (AMI) complicated by malignant ventricular arrhythmia (MVA) exhibited decreased plasma levels of TGFBI and increased levels of vWF, both of which can be candidate biomarkers for MVA. Reitz et al. (73) performed proteomic and phosphoproteomic analyses on left ventricular tissue from HF (HF) patients, and identified that the αT-catenin phosphorylation modifications were associated with the risk of DCM, and provided important insights into HF pathogenesis and precision treatment strategies. Commercial proteomics solutions such as SomaScan and Olink™ have significantly contributed to the field of biomarker discovery (74, 75). Helgason et al. developed a risk scoring model based on 70 proteins using the SomaScan v4 platform, which achieved a modest improvement of the prediction accuracy of atherosclerotic cardiovascular disease (ASCVD) risk (76). Through integrated proteomics and machine learning technologies, Nurmohamed et al. (77, 78) identified key protein biomarkers related to recurrent vascular events and constructed a risk prediction model that showed better performance compared to traditional clinical indicators. This achievement not only provides a more precise tool that will enhance the secondary prevention of CVDs but also opens new avenues for improving personalized nursing. However, accurate quantification and standardization of protein biomarkers are major technical challenges, and the relationship between protein expression and disease progression across different cardiovascular disease subtypes requires further investigation.

#### 2.1.5 Network medicine

Network medicine, as an interdisciplinary field, integrates systems biology and network science to explore interactions among biological components in health and disease states. It enables multi-omics data integration and analysis, allowing researchers to comprehensively understand CVDs (79, 80). Maron et al. (81) employed RNA sequencing and personalized protein-protein interaction network analysis on HCM patients and healthy controls. It was observed that network characteristics could effectively distinguish HCM from DCM, reflected differences in fibrosis phenotypes, and correlated closely with HF clinical phenotypes, offering critical insights for precision medicine in CVDs. Arif et al. (82) developed a mouse MI model and, through transcriptomics and network analysis, uncovered alterations in metabolic pathways during MI, providing new perspectives for tissue-specific therapeutic targets. Liu et al. (83) performed a comprehensive analysis on multi-omics data and identified that single nucleotide polymorphisms (SNPs) at the 9p21 locus were associated with MI risk, while proteins such as CK-MB served as diagnostic and prognostic biomarkers for MI. Furthermore, metabolites such as glutamine and glutamate were found to be critical indicators of the pathological state following MI. Wang et al. (84) investigated the pathogenesis of myocarditis and, through multi-omics data analysis, and identified that biomolecules such as TNF-α and IL-6 could influence the disease progression. Elsewhere, multi-omics network integration platforms such as Mergeomics 2.0 (85) were found to serve as online tools that could integrate disease-associated multi-omics data, uncovering disease-related pathways and their core regulatory factors, bridging the gap between molecular discovery and clinical application. These multi-omics network medicine (86) analytical methods and research platforms are expected to uncover biomarkers for cardiovascular diseases, advancing precision risk assessment, optimizing care interventions, personalizing drug therapy, and achieving more scientific and efficient individualized healthcare solutions.

### 2.2 Applications of AI in CVD care

Personalized healthcare has been a major topic in clinical research, and advancements in multi-omics technologies have led to the introduction of novel perspectives. However, the transition from genetic variations to personalized nursing remains a complex challenge. Wearable devices enable real-time collection of extensive biological data, which, when analyzed using AI algorithms, can yield in-depth insights and automatically generate treatment plans and clinical care recommendations. This approach overcomes current limitations in the field of personalized medicine (87, 88). AI technologies are widely applied in diverse data modalities and clinical scenarios, including β-blocker efficacy analysis, multi-omics data integration, medical text mining, early warning system development, wearable device data monitoring, vascular aging prediction, automated imaging analysis and interpretation, and the enhancement of nursing efficiency (89–91). his chapter provides an overview of the application scenarios of artificial intelligence (AI) in healthcare, highlighting its potential to advance personalized nursing through data-driven insights and precision decision-making (Table 2).

**Table 2:** Application of AI technology in cardiovascular nursing.

Disease type	Technology/Method	Application case	Results/Findings	Clinical significance	References
Improved diagnostic accuracy					
CVD risk prediction	ML	Various ML methods for CVD risk prediction	Random forest performed best for CVD risk prediction, with AUC significantly higher than other models	ML methods provide high accuracy in CVD prediction, especially with the random forest algorithm	Li Yang et al. (93)
Cardiovascular Disease Prediction	Automated Machine Learning (AutoPrognosis)	AutoPrognosis framework for CVD risk prediction, based on data from 423,604 UK Biobank participants	AutoPrognosis achieved an AUC-ROC of 0.774, outperforming the Framingham score (AUC-ROC = 0.724) and other conventional models	Provides more accurate CVD risk prediction, especially for subgroups where traditional models perform poorly (e.g., diabetes patients)	Ahmed M. Alaa et al. (94)
Cardiovascular Disease Diagnosis	CNN, RNN	AI analysis of cardiac ultrasound data to improve diagnostic accuracy	Deep learning models can analyze OCT images to identify early signs of CVD	Promotes non-invasive screening and enhances early diagnostic capabilities	Krittanawong et al., (95)
LVH Detection	CNN, ML	Detection of LVH using ECG	AI's SROC curve area: 0.87, Sensitivity: 69%, Specificity: 87%	AI shows higher diagnostic accuracy for LVH than traditional Sokolow-Lyon and Cornell criteria	Siranart et al. (99)
ACS (STEMI)	Cohen κ and Bland–Altman Analysis	Early diagnosis of STEMI using ECG data	SEN 93%, SPE 95%	Facilitates early STEMI identification and optimizes treatment timing	Spaccarotella et al. (100)
CAD Diagnosis	AI-QCT	CCTA analysis of coronary artery stenosis compared with invasive QCA	AI-QCT achieved AUC = 0.91 for detecting ≥70% stenosis, superior to experienced clinicians (AUC = 0.73–0.77)	AI-QCT provides higher diagnostic consistency for broad CAD patient groups, especially for less experienced clinicians	Bernardo et al. (101)
Personalized patient management					
Cardiovascular Disease	LLM	AI-assisted health management, including wearable data interpretation and follow-up	Improves patient convenience and adherence to health management	Potential for personalized health interventions and long-term cardiovascular health improvement	Ayers et al. (91)
Cardiovascular Risk Management	Mobile Health App, Ultrasound Imaging, Behavior Tracking	Mobile app for carotid plaque ultrasound imaging and lifestyle tracking	Patients using PreventiPlaque showed significantly lower CVD risk scores, with reductions in LDL cholesterol and blood pressure	Personalized health information and behavior tracking enhance patient engagement and adherence to CVD risk management	Ben Li et al. (106)
Personalized Preventive Medicine	Random Forest, SVM, Neural Networks, Big Data Analytics	Using EHR and wearable device data to predict CVD risk	Random forest performed best for CVD risk prediction (87% accuracy), followed by SVM and neural networks	AI-based personalized preventive medicine enables early intervention and CVD risk reduction	Nurani et al. (107)
HF Remote Monitoring	AI-assisted Diagnosis and Prediction	AI algorithms for HF event prediction	Early identification of HF deterioration risk, reducing hospitalization rates	Improves HF management efficiency and reduces healthcare costs	Kotalczyk et al. (108)
Remote Monitoring	Sensors, Machine Learning Algorithms	Wearable devices monitoring heart rate, blood pressure, and AI-based health analysis	Provides real-time health data, enhancing patient self-management	Early detection of cardiovascular abnormalities, reducing sudden cardiac events	Sethi et al. (104)
AMI	Wearable Devices + Smartphone Apps	Integrated smartwatches, blood pressure monitors, and mobile apps for AMI recovery	DHI reduced 30-day readmission rates (6.5% vs. 16.8%), saving $7,319 per patient	Demonstrates the value of DHI in long-term cardiovascular disease management	Marvel et al. (105)
Optimized clinical workflow					
Aortic Stenosis (AS) Detection	Deep Learning Models	Automatic Detection of Severe AS Using Single-View 2D TTE Videos	The model demonstrated high diagnostic performance across geographically and temporally diverse cohorts (AUROC: 0.942–0.978).	Provides an automated screening tool that does not require Doppler imaging, making it suitable for resource-limited healthcare settings.	Holste et al. (114)
ECHO	Deep Learning	AI-guided ultrasound imaging by non-experts, improving image quality.	AI enhances image quality, expanding ECHO applications in primary care.	Promotes precise imaging diagnostics and broadens ECHO accessibility.	Narang et al. (115)
AI-Assisted Diagnosis	Image Recognition, Neural Networks	AI interpretation of cardiac MRI, echocardiography, CT scans, and ECG.	Enhances diagnostic accuracy.	Reduces physician workload and improves diagnostic efficiency.	de Marvao et al. (118)
Physician Assistance	Computer-Aided Decision Systems	AI-assisted analysis of medical records and literature.	Improves physician decision-making and reduces human errors.	Accelerates diagnosis and treatment decisions, enhancing healthcare quality.	Gallone et al. (119)
Cardiovascular Imaging and Functional Assessment	AI + Coronary CT Angiography (CCTA)	AI-assisted CCTA for plaque volume assessment compared to traditional methods.	AI improves diagnostic confidence and reduces unnecessary testing (37.1% reduction).	Enhances imaging accuracy and efficiency, optimizing clinical decision-making.	Nurmohamed et al. (120)
Early intervention and prevention					
MI Prediction	Deep Learning Models	Prediction of MI risk.	Deep learning models outperform traditional methods (AUC = 0.835).	Early identification of high-risk patients, optimizing preventive strategies.	Mandair et al. (123)
Multi-Label Electrocardiogram (ECG) Diagnosis	Deep Learning (CNN)	Simultaneous Diagnosis of Multiple Arrhythmias via ECG Analysis	The multi-label diagnosis achieved an F1-score of 0.887, outperforming physicians (0.789–0.831).	Enhances the accuracy of complex arrhythmia diagnosis, optimizing treatment strategies.	Zhu et al. (124)
LVH Detection	Deep Learning Models	Automated Detection of Left Ventricular Hypertrophy and Its Subtypes via Echocardiography	The model demonstrated high precision in detecting left ventricular hypertrophy and its subtypes.	Improves the diagnostic efficiency of left ventricular hypertrophy, facilitating early intervention.	Grant et al. (125)
Atrial Fibrillation Prediction	CNN	Prediction of atrial fibrillation using wearable ECG monitors.	AI-ECG detects AF with AUC = 0.87, SEN = 79%, SPE = 79.5%, ACC = 79.4%.	Early identification of high-risk AF patients, facilitating timely treatment.	Attia et al. (126)
CAD Risk Screening	Deep Learning Models	AI detection of CAC in non-ECG-gated chest CT for ASCVD risk assessment.	AI detects CAC with high sensitivity (82%–94%) and positive predictive value (87%–100%).	Enables early screening and intervention, reducing cardiovascular events.	Eng et al. (127)
Major Adverse Cardiovascular Events (MACE)	AI Model Analysis of Chest x-rays	AI analysis of 8,869 chest x-rays compared to traditional ASCVD risk scores.	AI-predicted MACE risk more accurate (HR = 1.73, 95% CI: 1.47–2.03).	Potential for non-invasive risk assessment and personalized treatment optimization.	Weiss et al. (128)

#### 2.2.1 Enhancing diagnostic accuracy

AI models have shown great potential to improve the diagnosis of CVD, particularly in detecting arrhythmias, HF, and MI, with significant advantages over conventional diagnostic methods. Numerous studies have employed machine learning models to predict CVD risk (92). For instance, a study on about 30,000 participants found that a random forest model could accurately predict the three-year CVD progression relative to traditional multivariate regression models (93). Similarly, an analysis of data from over 400,000 participants in the UK Biobank uncovered that machine learning-based pipelines outperformed the Framingham risk score and Cox proportional hazards model in predicting CVD risk (94). Moreover, the convolutional neural networks (CNNs) and deep neural networks (DNNs) were employed to analyze electrocardiographic (ECG) data to detect arrhythmia. These tools showed high precision performance, enabling the accurate identification of abnormal cardiac rhythms (95). Left ventricular segmentation technology allows effective management of cardiovascular diseases. Notably, deep learning-based cardiac MRI/CT left ventricular segmentation techniques enables automatic quantification of key functional parameters, such as ejection fraction, and the precise measurement of myocardial mass and detection of local ventricular wall motion abnormalities (96–98). AI has also shown notable advantages in diagnosing left ventricular hypertrophy (LVH). Compared to conventional Sokolow-Lyon and Cornell criteria, AI models incorporating deep learning (CNN) and machine learning (ML) techniques which offer higher sensitivity and specificity, and hence earlier and more accurate LVH detection (99). Spaccarotella et al. reported that multi-lead ECG recordings obtained from smartwatches (e.g., Apple Watch Series 4) exhibited strong concordance with standard ECGs in detecting ST-segment changes in patients with ACS, suggesting that AI-assisted wearable technology can achieve early ACS detection (100). Moreover, in the comparison of AI-based quantitative coronary tomography (AI-QCT) with clinical coronary CT angiography (CCTA) assessments performed by physicians of varying expertise levels, it was observed that AI-QCT had better prediction consistency with invasive quantitative coronary angiography (QCA) in detecting obstructive coronary artery stenosis, outperforming standard CCTA assessments. Notably, the integration of AI-QCT in clinical practice can potentially enhance the reproducibility and reliability of CCTA evaluations (101).

Collectively, these findings indicate that AI can optimize the diagnostic precision and facilitate personalized treatment recommendations, enabling healthcare professionals to optimize cardiovascular disease management, minimize unnecessary diagnostic procedures and interventions, and ultimately improve patient outcomes and quality of life.

#### 2.2.2 Personalized patient management

AI-driven wearable devices and remote monitoring systems offer innovative solutions for personalized patient management. These technologies enable real-time acquisition of physiological parameters, such as heart rate, blood pressure, and ECG readings, which can be analyzed using AI algorithms to provide tailored health recommendations and therapeutic interventions (102–105). Ullrich et al. developed a smartphone-based AI application, PreventiPlaque, that integrates carotid plaque ultrasound imaging to quantify plaque burden. This application could effectively monitor the progression of atherosclerotic disease while tracking physical activity, dietary habits, medication adherence, and smoking status. The app employs goal-setting and interactive visual cues to encourage lifestyle modifications, which improves cardiovascular health (106). Nurani et al. integrated real-time data from wearable technologies (e.g., smartwatches and health trackers) to continuously monitor cardiovascular parameters such as heart rate, blood pressure, and glucose levels, enabling dynamic intervention adjustments (107). The AI-based systems can modify pharmacological regimens or recommend lifestyle modifications based on real-time blood pressure readings, thereby enhancing intervention timeliness and improving patient adherence. A previous investigated explored the performance of remote monitoring technologies incorporating implantable devices, such as the CardioMEMS™ system (108). They found that the devices enabled continuous pulmonary artery pressure (PAP) monitoring (19). This technology facilitates early detection of hemodynamic changes, reducing hospital readmission rates and improving the quality of life in patients with HF (109, 110). A randomized controlled trial (RCT) further demonstrated that AI-assisted remote monitoring significantly improved HF patients’ quality of life and reduced rehospitalization rates (104, 111). AI- and big data-driven personalized health plans have demonstrated a 90% patient adherence rate, compared to 65% for conventional management strategies. These AI-based interventions can improve cardiovascular health by 25%, reduce diabetes-related complications by 30%, and decrease healthcare costs by 20% (107). AI-driven systems not only enhance patient self-management, but also provide clinicians with comprehensive and accurate health data, enabling precision medicine in cardiovascular care.

#### 2.2.3 Optimizing clinical workflow

AI has been shown to optimize clinical workflows by automating imaging analysis, ECG interpretation, and other repetitive tasks, thereby reducing the burden on healthcare professionals and allowing them to focus on complex decision-making processes. In cardiovascular imaging, AI algorithms can efficiently and accurately process echocardiography, cardiac MRI, and CT scan data (112, 113). AI-assisted deep learning systems have been shown to rapidly identify Aortic stenosis (AS) severity characteristics, providing diagnostic support for clinicians (114). Deep learning algorithms also enable non-expert healthcare personnel to acquire high-quality echocardiographic images, broadening the accessibility of echocardiography in clinical practice (115). Machine learning algorithms further facilitate automated quantification of cardiac parameters, detection of cardiovascular pathologies, and assessment of cardiac function, which improves the diagnostic accuracy and efficiency for diseases (116, 117). AI-driven solutions can automate cardiovascular imaging analysis, predict patient survival, monitor arrhythmias, assist in interventional and electrophysiological procedures, and enhance diagnostic precision, efficiency, and safety (118, 119). Studies have shown that AI-based algorithms can optimize rapid ECG data analysis, identify arrhythmias and ischemic abnormalities while generating detailed diagnostic reports. Physicians should review and validate AI-generated results, thereby shorten the interpretation time and workload (120). Although AI has significantly improved real-time liver segmentation and other fields, its actual application in cardiovascular diagnosis and treatment faces several challenges such as computational efficiency, cross-modal generalization ability and clinical integration (121, 122).

Studies have demonstrated that the deep integration of AI with cardiovascular clinical workflows enhances the quality and efficiency of medical services. However, this process requires the collaborative efforts from clinicians, engineers, and regulatory authorities, to create an optimal balance in terms of technical performance, ethical norms, and clinical application, thereby achieving an intelligent transformation of the cardiovascular diagnosis and treatment model.

#### 2.2.4 Early intervention and prevention

AI has demonstrated high potential to identify high-risk patients, facilitate early intervention and prevent CVDs. By enabling timely risk assessment, AI-driven predictive models can guide preventive strategies, minimizing the risk of complications and improving long-term patient outcomes. Mandair et al. utilized machine learning algorithms to analyze EHR for MI prediction, and developed a novel approach for cardiovascular risk stratification (123). Zhu et al. employed deep learning models for automated ECG interpretation that comprised arrhythmia classification, multi-label ECG diagnostics, atrial fibrillation detection, and ventricular tachycardia identification. These AI-based methods offer enhanced analytical accuracy and personalized treatment recommendations while minimizing human error (124). Grant et al. (125) developed a deep learning-based automated LVH detection tool, which facilitated accurate and early identification and differentiation of LVH etiologies. Atrial fibrillation, a frequently underdiagnosed arrhythmia, can be effectively detected using AI-ECG models based on CNNs, as reported by Attia et al. These AI-driven models offer rapid and cost-effective screening, enabling timely intervention (126). AI-assisted automated coronary artery calcium (CAC) scoring was reported to enhance the detection of coronary calcifications in routine chest CT scans, providing an opportunity for early cardiovascular risk mitigation (127). Furthermore, AI applications in risk stratification can be applied to chest x-ray analysis to enable cardiovascular disease detection. Weiss et al. (128) demonstrated that AI-driven models effectively predicted major adverse cardiovascular events, presenting good potential for incorporation in cardiovascular care.

## 3 Application of personalized nursing in CVDs

Personalized medical care not only enhances patient health by facilitating precise diagnosis, prevention, and treatment, but also can be tailored based on individual characteristics, contexts, backgrounds, and environments to support self-management and independent living (129, 130). Table 3 summarizes the impact of physiological, psychological, social, and cultural factors on CVDs and proposes personalized nursing strategies for each patient.

**Table 3:** The impact of physiological, psychological, social and cultural levels on personalized cardiovascular nursing.

Category	Application case	Significance of personalized nursing	References
Physiological			
HF	The heart is unable to pump blood effectively, leading to fluid accumulation; maintaining volume balance is a key factor in managing HF.	Strict monitoring of fluid balance, low-sodium diet, standardized medication use, symptom recognition, and lifestyle adjustments.	Cosentino, N. et al. (134)
Arrhythmia (Atrial Fibrillation)	Atrial fibrillation may be associated with abnormal cardiac electrophysiological activity, which can be influenced by electrolyte imbalances (e.g., hypokalemia) and physiological factors such as thyroid dysfunction.	Identifying potential risk factors for atrial fibrillation to prevent further exacerbation of arrhythmia.	Brundel, B. et al. (137)
Hypertensive Heart Disease	Due to the prolonged high workload on the heart, cardiac function is impaired, making patients prone to fatigue, dyspnea, and reduced exercise tolerance.	Strict blood pressure control, standardized medication use, lifestyle optimization, cardiac function monitoring, and psychological support.	Díez, J. and J. Butler (140)
Disease Severity (Mild Cases)	Early-stage heart disease (HF Stage A & B)	Healthy lifestyle (regular exercise, smoking cessation, healthy diet); Use of multivariable risk scores to assess HF risk.	Heidenreich, P.A. et al. (143)
Disease Severity (Severe Cases)	Late-stage heart disease (HF Stage C & D)	Non-pharmacological interventions: Multidisciplinary team management; Sodium intake restriction; Regular exercise training or cardiac rehabilitation program. Pharmacological interventions: HFpEF patients: Blood pressure control, SGLT2 inhibitors.	Heidenreich, P.A. et al. (143)
Gender Differences	Men's renal clearance rates are typically higher than women's; sex differences in pharmacodynamics may include increased or decreased drug effects and adverse reactions.	Women may be more sensitive to certain medications; treatment plans should consider female physiological characteristics and drug metabolism differences to achieve personalized therapy.	Mensah, G.A. and V. Fuster. (149)
Aging and Menopause	Middle-aged women experience metabolic, physiological, and psychosocial changes during menopause, negatively affecting overall quality of life.	Aerobic exercise can enhance NO bioavailability and improve vascular function.	Nobuaki Fukuma et al. (151)
Elderly Cardiovascular Patients	With aging, increased oxidative stress and chronic inflammation can contribute to cardiovascular disease.	Regular physical activity is crucial for preventing cardiovascular disease in older adults, improving physiological parameters, reducing disease risk, and enhancing quality of life.	Lorena Ciumărnean et al. (153)
Genetic Factors	Multiple RCTs and real-world studies support gene-guided therapy to reduce adverse event risks.	Genetic testing can guide cardiovascular drug selection, significantly improving treatment safety and efficacy.	Julio D Duarte et al. (155)
Psychological			
Psychological Stress	Research shows that psychological stress is associated with increased cardiovascular disease risk, particularly hemodynamic, vascular, and immune system disturbances.	Psychological stress assessments should be incorporated into clinical care and prevention, with stress management interventions potentially reducing cardiovascular risk.	Vaccarino et al. (156)
Negative Mental Health	Depression, anxiety, chronic stress, and PTSD are significantly associated with increased cardiovascular disease risk.	Negative mental health is an important cardiovascular disease risk factor; clinical screening and interventions are needed.	Commodore-Mensah et al. (160)
Anxiety Disorders	Studies show significant causal relationships between anxiety disorders and CHD (OR: 4.496, *P* = 0.002), MI (OR: 5.042, *P* = 0.011), and HF (OR: 3.255, *P* = 0.004).	Anxiety disorders increase the risk of CAD, MI, and HF, necessitating strengthened mental health management to reduce cardiovascular disease incidence.	Bo Peng et al. (161)
Social Isolation & Loneliness	A cohort study of 57,825 elderly women in the U.S. found associations between social isolation, loneliness, and CVD.	Implementing interventions to reduce social isolation and loneliness in this population.	Natalie M Golaszewski et al. (167)
Social			
Economic Factors	SES is associated with higher 10-year ASCVD predicted risk; SES indicators correlate with CVD risk.	CVD prevention strategies should consider socioeconomic status (SES) factors.	Alexandre Vallee et al. (171)
Shift Work	shift work is associated with an increased risk of CVD, increasing CVD event risk (RR 1.17, 95%CI 1.09–1.25) and CVD mortality (RR 1.22, 95%CI 1.09–1.37).	Shift work is significantly associated with cardiovascular disease risk, particularly for long-term shift workers.	Torquati et al. (176)
Physical Labor	Intense OPA and CVD mortality exhibit a U-shaped relationship, with both no intense OPA and high-intensity OPA workers having higher CVD mortality than low-intensity OPA workers.	Workload balance and cardiovascular health monitoring should be implemented to reduce CVD mortality risk.	Tong Xia et al. (178)
Air Pollution	Major pollutant PM2.5 is closely related to hypertension; chronic exposure to air pollution, even at low levels, increases cardiovascular disease morbidity, hospitalization, disability, mortality, and costs.	Emphasizing air pollution as a modifiable factor contributing to cardiovascular disease burden and public health costs, providing a basis for preventive measures.	Khoshakhlagh et al. (188)
Climate Change	Climate change increases cardiovascular disease incidence and mortality through extreme temperatures, air pollution, and extreme weather events.	Climate change is a significant cardiovascular disease risk factor; countermeasures should be strengthened.	Khraishah et al. (187)
Cultural			
Language & Communication Styles	Language barriers impact access to health resources, and cultural beliefs influence self-care behaviors.	Language barriers affect health information access and service utilization; families play a crucial role in self-care and should be included in health education programs.	Ling Zeng et al. (195)
Traditional Dietary Culture	Higher dietary cultural adaptation scores (i.e., closer to mainstream American diets) are significantly associated with increased CVD risk (HR = 1.33, 95% CI: 1.13–1.57).	Integrating culturally sensitive dietary guidance can more effectively reduce CVD risk among immigrant populations, promoting precision and personalized health management.	Yi Wang et al. (191)
Race	Race and racism affect cardiovascular health through complex social, economic, environmental, and healthcare system pathways.	Personalized nursing should go beyond traditional biomedical models, integrating social determinants to address health disparities through systemic reforms and targeted interventions.	Zulqarnain Javed et al. (192)
Religion & Spiritual Beliefs	Studies indicate a positive correlation between religious beliefs and spirituality (R/S) and medication adherence, such as religious activities and prayer improving adherence among hypertensive patients.	Designing spiritually integrated intervention strategies based on patients’ religious and cultural backgrounds to improve treatment adherence and health outcomes.	Marwa Elhag et al. (197)

### 3.1 Physiological factors

Clinically, CVDs constitute a broad spectrum of conditions, each with distinct pathophysiological mechanisms and clinical manifestations. Therefore, implementing personalized nursing requires a comprehensive assessment of patients' physiological information, including medical history review, physical examinations, laboratory tests, and imaging data analysis (131).

For instance, CHF is caused by impaired cardiac pump function, which leads to fluid retention, which prolongs disease progression and hospital readmission (132, 133). Thus, effective volume management is critical in controlling CHF, requiring a multidimensional intervention approach that includes strict fluid balance monitoring, a low-sodium diet, standardized medication use, symptom recognition, and lifestyle adjustments (134–136). Patients with arrhythmias, particularly atrial fibrillation, present with electrophysiological abnormalities associated with electrolyte imbalances (e.g., hypokalemia) and thyroid dysfunction (137, 138). In personalized nursing assessments, it is imperative to identify low serum potassium levels to mitigate the risk of arrhythmias. Hypertensive heart disease (HHD), a condition caused by prolonged hypertension, is associated with structural and functional cardiac alterations (139, 140). It is often managed through a combination of strategies, including strict blood pressure control, medication adherence, lifestyle optimization, cardiac function monitoring, and psychological support meant to delay cardiac remodeling and reduce HF risk.

Nursing strategies are implemented based on the disease severity. Patients with mild symptoms can benefit from early-stage interventions, such as health education and lifestyle modifications, achieving optimal blood pressure control and reducing medication dependency in some patients (141). In contrast, severe cases require intensive nursing care, including 24-hour electrocardiographic monitoring for detecting arrhythmias, stringent fluid intake and output control, meticulous bedside care to prevent pressure ulcers and thrombosis. Based on the New York Heart Association (NYHA) classification, patients with Class II HF can benefit from tailored physical activity guidance to enhance myocardial reserve capacity (142). Meanwhile, for Class IV patients, strict sodium intake restrictions, precise fluid management, and pharmacological interventions should be applied to alleviate cardiac burden (143–145).

Physiological and genetic factors need to be incorporated in personalized nursing. Studies indicate that sex differences influence cardiovascular drug responses, with males generally exhibiting higher renal clearance rates, while females presenting with variations in pharmacodynamics, including enhanced drug efficacy, diminished effects, or increased adverse reactions (146–149). Vascular changes in women associated with aging and menopause, such as arterial stiffness and endothelial dysfunction, can be mitigated through aerobic exercise, which enhances nitric oxide (NO) signaling and improves vascular function (150, 151). For elderly patients with CVDs, personalized dietary and exercise interventions significantly improve symptoms—particularly in HF—while optimizing blood pressure, lipid profiles, and insulin sensitivity (152, 153). Genetic predisposition is a major predictor of cardiovascular disease risk, accounting for approximately 50% of disease susceptibility. Advances in genetic testing have led to the formulation of pharmacotherapy based on genetic profiles, with CYP2C19 genotyping commonly used to predict clopidogrel responsiveness and guide antiplatelet therapy following percutaneous coronary interventions (154, 155). Such genetic-based interventions improve therapeutic outcomes while reducing adverse effects and economic burdens.

Notably, the application of personalized physiological nursing interventions is based on disease type, severity, physical function, and genetic traits, enabling selection of precise interventions that effectively alleviate symptoms, control disease progression, and minimize complications, enhancing the overall patient health.

### 3.2 Psychological factors

Studies have shown that CVDs affect the physical health as well as various psychological challenges. Chronic psychological stress, occupational stress, depression, perceived discrimination, loneliness, and social isolation are prevalent among cardiovascular patients, significantly affecting treatment adherence and recovery outcomes. A strong association has been reported between psychological stress and elevated cardiovascular disease risk (156, 157). Acute stress, although transient, can exacerbate existing cardiovascular conditions and trigger adverse cardiac events. In contrast, chronic stress promotes the progression of atherosclerosis, increasing the risk of CVDs (158). Psychological stress has been linked to the development of mental health disorders such as depression and post-traumatic stress disorder (PTSD), both of which elevate cardiovascular risk (159–161). Research indicates that depressive symptoms are strong risk factors of CAD, MI, and HF (158, 162, 163). Position statements from the American Heart Association and the European Society of Cardiology recognize depression as a modifiable prognostic factor in CAD and advocate for its early identification and management (164, 165). In a cohort study of 57,825 older women in the U.S., those with higher levels of social isolation and loneliness had a 13.0%–27.0% increased risk of CVDs compared to their less isolated counterparts (166, 167).

Of note, implementation of psychological interventions in nursing care has been suboptimal owing to the varying levels of expertise among healthcare providers, which calls for further training to enhance psychological nursing competencies. Moreover, given the lack of standardized, precise assessment tools for evaluating psychological interventions, further research is advocated to develop robust evaluation frameworks.

### 3.3 Social factors

Social determinants of health (SDOH) refer to a wide range of environmental and societal factors which influence health outcomes, including income, education, employment, social support, and living conditions (168, 169). Favorable social conditions can protect cardiovascular health, while upstream SDOH factors interact across domains to exert cumulative downstream effects on physiological and behavioral pathways (170).

Economic status often influence the choice of treatment options and rehabilitation processes for cardiovascular patients (171). Findings from large-scale population studies indicate that individuals from impoverished communities have a 1.3–1.8 times higher risk of developing MI compared to those from high-income areas (172). Moreover, individuals of lower socioeconomic status who experience early-onset heart disease have a 32% higher long-term mortality rate than their higher-status counterparts (173). Thus, providing financial assistance via medical aid and selecting cost-effective treatment options can alleviate financial burden and ensure continuity of care. Occupational factors can also significantly affect cardiovascular rehabilitation and reintegration into society (174). Studies have linked shift work to increased cardiovascular disease risk, particularly among long-term shift workers, as it contributes to hypertension, dyslipidemia, metabolic syndrome, and impaired glucose metabolism, elevating the risks of cardiovascular disease and type 2 diabetes (175–177). In a questionnaire survey involving 28,604 people in the United States investigating the association between occupation and cardiovascular disease, a U-shaped relationship was observed between occupational physical activity (OPA) and CVD mortality, and this relationship was influenced by socioeconomic status (SES) (178).

Environmental stressors, such as air pollution, noise exposure, and artificial nighttime lighting, influence the risk of non-communicable diseases, including CVDs (179). A meta-analysis found that traffic noise exposure elevates the risk of cardiovascular disease risk (180). This calls for interventions to control noise and air pollution to mitigate cardiovascular health risks (181, 182). Unhealthy environments have been linked to a 25% increase in the risk of ischemic heart disease, with air pollution significantly contributing to disease prevalence, hospitalizations, disabilities, mortality, and healthcare costs (183). Given the direct and indirect impact of climate change on cardiovascular health, particularly in vulnerable populations, multidisciplinary and multi-level strategies need to be developed to address SDOH challenges affecting cardiovascular care (184–187). In the future, researchers should investigate the risk factors of air pollution and propose interventions to alleviate the burden of CVDs (185, 188).

### 3.4 Cultural factors

Cultural dimensions can influence disease perception, treatment adherence, and health outcomes (189). Understanding patients' cultural backgrounds, including dietary habits, religious beliefs, and traditional practices, fosters a respectful and supportive care environment, which is crucial to patient satisfaction and improving overall health outcomes (190).

For example, Yi Wang's study (191) demonstrated that dietary cultural adaptation among Hispanic and Latino adults in the U.S. increased the risk of cardiovascular disease, which was potentially mediated by alterations in gut microbiota composition. Similarly, a study by Zulqarnain Javed (192) uncovered that racism was an important factor influencing cardiovascular health via behavioral and physiological pathways, such as stress-induced inflammation. The incidence and mortality associated with cardiovascular disease vary between immigrant populations and host populations, which are attributed to lifestyle changes, psychosocial stress, and socioeconomic disadvantages following migration (193–195). Religious beliefs and spirituality also play a critical role in shaping health behaviors among cardiovascular patients. For instance, in Ghana, many hypertensive patients prioritize “Divine Healing” over evidence-based treatments due to strong religious convictions (196, 197). Culturally and linguistically adapted educational interventions have shown promise in improving disease knowledge, promoting self-care behaviors, and reducing hospitalizations (198). Furthermore, culturally sensitive nursing practices—such as respecting cultural norms, optimizing communication strategies, and tailoring interventions to individual preferences—improve patients' trust, treatment adherence, and ultimately promote holistic health outcomes.

## 4 Clinical significance of combining precision medicine and personalized nursing

**Figure 1:**
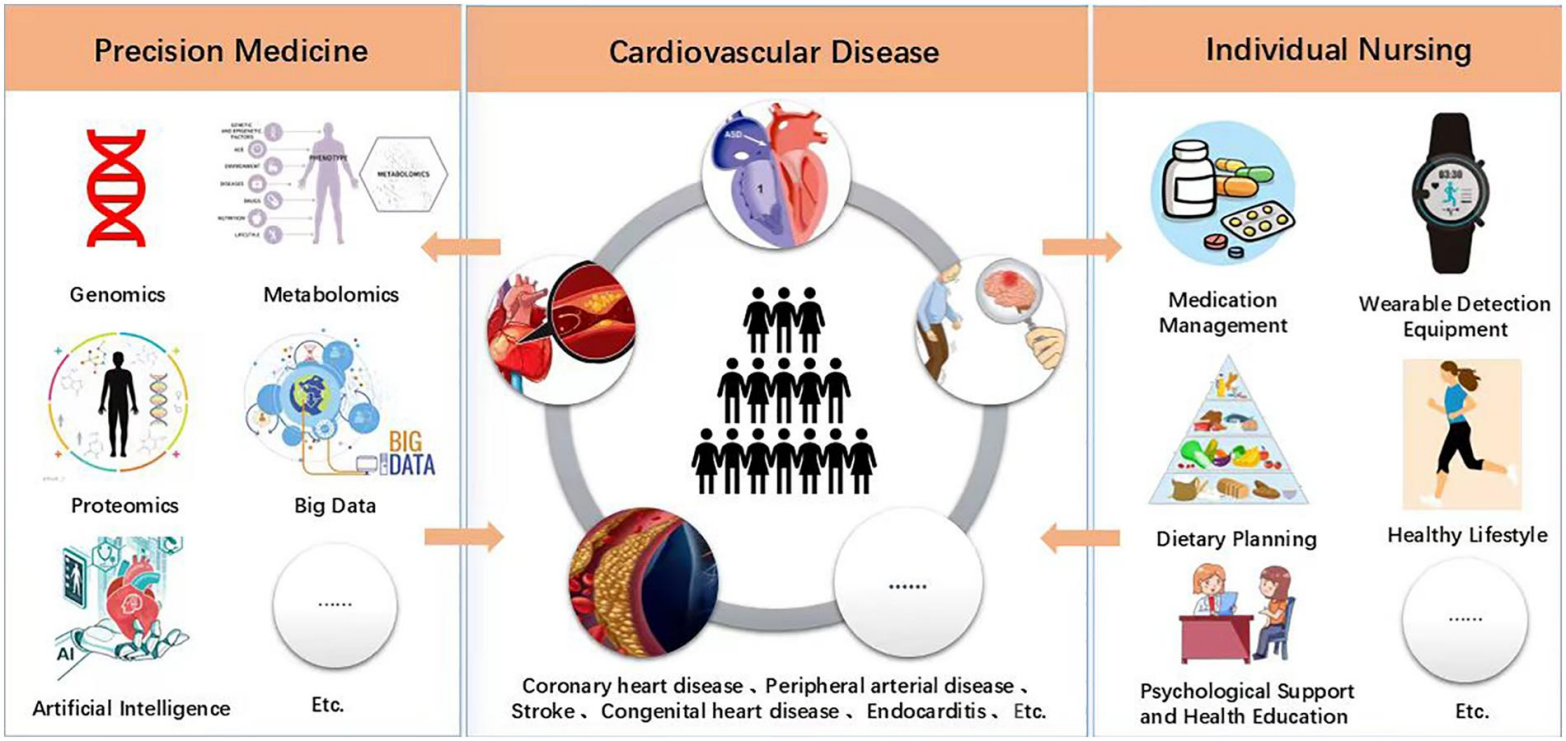
The connection between precision medicine and personalized nursing.

Integrating precision medicine and personalized nursing not only includes patients' physiological monitoring data but also non-physiological indicators such as psychological, social, and cultural factors. This leads to the development of a more comprehensive “Patient Holistic Profile” for healthcare professionals. Moreover, the integration of multidimensional information enables medical teams to make more accurate and informed clinical decisions. Precision medicine and personalized nursing complement are complementary approaches in terms of concepts, methods, and practical applications. Their common goal is to improve patient treatment outcomes and quality of life, but the core distinction lies in their focus: personalized nursing emphasizes the implementation of comprehensive and customized care plans based on individual patient characteristics (such as genetic background, lifestyle, and social factors), while precision medicine uses high-throughput technologies such as genomics and proteomics to finely subtype patients at the molecular level, categorizing diseases into subgroups and developing targeted treatment plans to achieve early diagnosis and precise intervention (199, 200). Critically, the success of this integrated model relies on patient stratification, which identifies individuals most likely to benefit from specific interventions while minimizing unnecessary treatments. Stratification strategies may include the use of PRS to quantify inherited disease susceptibility and prioritize preventive measures; phenomapping through unsupervised machine learning applied to high-dimensional EHR and clinical data to identify actionable subphenotypes within heterogeneous conditions [e.g., heart failure with preserved ejection fraction (HFpEF)] and differentiate likely “responders” from “non-responders”; and biomarker-guided selection (e.g., NT-proBNP or inflammatory markers) to refine patient eligibility and inform individualized dosing and monitoring protocols. Therefore, the combination of both approaches (Figure 1) not only optimizes therapeutic effects but also promotes a shift in nursing models from experience-driven to data-driven, making nursing more scientific and precise, while significantly enhancing patient satisfaction and treatment adherence (201).

Specifically, the integration of precision medicine and personalized nursing has several advantages: First, it can enhance the accuracy and specificity of nursing care. The combination of precision medicine and personalized nursing may generate personalized nursing plans which are suited to the specific features of individual patients, making nursing more precise and effective. For example, cardiovascular disease patients with different genotypes may exhibit variations in drug metabolism and response; precision medicine can use genetic testing techniques to select the most suitable drugs and dosages for patients, improving treatment efficacy and reducing adverse drug reactions (202, 203). Another notable advantage is that personalized nursing provides tailored psychological support, health education, and rehabilitation guidance based on patients' psychological states, lifestyles, and social backgrounds, further enhancing patient compliance with treatment and quality of life (204). Second, this approach fosters patient trust, thereby enhancing treatment satisfaction and adherence. By recognizing that care plans are individually tailored, patients demonstrate greater engagement, motivation, and willingness to participate in their treatment. Third, from an economic perspective, the combined model of precision medicine and personalized is superior to traditional nursing models in terms of medical costs (including medication costs, examination fees, and hospitalization expenses) and health outcomes (such as quality-adjusted life years and benefits derived from reduced disease recurrence rates). For instance, it was reported that personalized nursing models are more cost-effective than conventional nursing models, with probabilities of 80%–99% over two years and 75%–90% over five years, demonstrating lower costs and better therapeutic effects (205). Finally, the application of this personalized framework can significantly enhance the prevention and control of cardiovascular diseases, as well as improve the efficiency of medical resource utilization. By accurately identifying high-risk populations through patient stratification tools, this approach reduces the number needed to treat (NNT), minimizes patients' exposure to unnecessary pharmacological toxicity, and effectively lowers the population-level incidence of cardiovascular diseases, while simultaneously optimizing the allocation of healthcare resources (206). This integration is expected to promote the development of the healthcare industry and professional talent cultivation, enhancing interdisciplinary integration among medicine, biology, and information technology, fostering the development of multidisciplinary medical professionals.

## 5 Discussion

Cardiovascular medicine is experiencing a paradigm shift from experience-based practice to precision medicine, owing by the emergence of three fundamental pillars: omics technologies, AI, and personalized nursing. Among them, omics technologies reveal multidimensional molecular insights into disease mechanisms, AI allows intelligent enhancement of diagnostic and therapeutic workflows, and personalized nursing provide comprehensive management systems centered around the patient. The integration of these elements heralds a new era characterized by precise, efficient, and individualized prevention and treatment strategies for cardiovascular diseases.

The application of omics technologies in cardiovascular precision medicine has progressed from single-dimensional studies to systematic multi-omics integration, revealing important molecular mechanisms and accelerating the clinical translation of new technologies. Genomic studies have uncovered several genetic markers underlying the pathology of pulmonary arterial hypertension and cardiomyopathies, which have been used to design polygenic risk score with prediction accuracies as high as 40% (46, 47, 51). For instance, the use of single-cell transcriptomics has unveiled key cardiomyocyte heterogeneity and identified novel biomarkers such as SEMA4C (56). Metabolomics have improved our understanding of the quantitative relationships between inflammatory markers and ventricular function, whereas proteomics has enabled disease subtyping and the discovery of post-translational modifications through high-precision platforms. Despite challenges related to sample heterogeneity and data standardization, network medicine integration has facilitated the development of comprehensive maps that facilitate molecular interactions, thereby laying the groundwork for developing targeted therapies. In future, researchers need to focus on dynamic multi-omics risk assessment systems and network medicine-based multi-target intervention strategies to stimulate the translation from molecular mappings to clinical applications.

AI technologies have created a closed-loop management system that spans from risk early warning to precision diagnosis and treatment, which has fundamentally improved cardiovascular disease prevention and management. In diagnostic applications, AI models have shown a 98% accuracy in detecting arrhythmia, outperforming even experienced cardiologists (100, 124). AI algorithms incorporated in smartwatches promote early detection of acute coronary syndromes, and have expanded the diagnostic window by 3–5 h (100). Regarding treatment management, AI-enhanced implantable devices can decrease the rates of heart failure readmission by 35%, while big data-driven personalized treatment regimens can improve patient adherence from 65% to 90% (107, 109). It has reported that AI-powered automated quantification can improve echocardiography efficiency by 50% and shorten the ECG interpretation time by 80% (112, 115, 124). Collectively, these advancements establish an intelligent closed-loop system that combines risk prediction with precision intervention. However, several challenges remain, including the “black box” nature of AI systems (such as issues of model interpretability and liability in AI-assisted diagnosis), reliance on high-quality annotated data, limited generalizability across real-world clinical settings (including model performance stability across novel scenarios, devices, diverse populations, and disease stages), and the potential for algorithmic bias (leading to diagnostic disparities due to overrepresentation of certain demographic groups in training data). This calls for the development of strategies aimed at resolving these limitations and accelerating the integration of AI in the entire cardiovascular care system.

Personalized nursing provides an important strategy for improving cardiovascular disease management owing to its capacity to integrate physiological monitoring with psychological, social, and cultural factors into precision care plans. Differential nursing strategies based on NYHA classification and pharmacogenomics reduce readmission rates by 25%–30% (143, 155). Psychological interventions help lower stress-associated cardiovascular risks by 13%–27% (167). Economic support programs for low-income populations help reduce health disparities, lowering myocardial infarction risk from a 1.8-fold to a 1.2-fold increase compared to higher-income groups (173). Culturally adapted strategies, such as dietary modifications for Latino immigrants, improve treatment adherence by 40% (191). The latest breakthroughs in personalized cardiovascular care stem from integrated, multidimensional assessment frameworks that combine NYHA classification, pharmacogenomics, and social determinants of health (SDOH) into a unified, closed-loop management system—spanning precision diagnosis to culturally tailored treatment plans. Looking ahead, the development of smart sociocultural screening tools and the establishment of multi-tiered collaborative networks linking.

Future studies should focus on three key aspects: the clinical implementation of dynamic multi-omics risk assessment systems, optimization of AI-driven clinical decision support systems, and the development of intelligent sociocultural screening tools. Achieving these goals requires the formulation of multidisciplinary collaborative networks that include healthcare, research, industry, and policy sectors to address systemic challenges such as data silos, algorithmic bias, and health inequities. The incorporation of these technologies is likely to improve cardiovascular medicine and accelerate the change from molecular mechanisms to clinical practice, ultimately realizing the “4P” vision of precision medicine: Predictive, Preventive, Personalized, and Participatory.

## 6 Conclusions

Personalized nursing have diverse clinical effects in cardiovascular care, and can improve patients' physiological conditions as well as their mental health and quality of life. Through precise symptom monitoring, individualized drug management, nutritional interventions, and exercise programs, personalized nursing provides comprehensive which is customized to the specific needs of each patient, thereby reducing the incidence of complications and improve treatment adherence. Future research should develop more efficient multi-omics data integration methods, enhancing the interpretability and clinical applicability of AI algorithms that will optimize precision medicine and personalized nursing in resource-limited areas, and establishing interdisciplinary collaboration platforms to promote the translation of basic research into clinical practice.

However, the integration of cardiovascular personalized nursing and precision medicine faces presents significant challenges, including multi-modal data integration, algorithmic bias in predictive models, and data privacy and security concerns. Currently, its clinical application is still in its infancy (89, 207, 208). First, fairness and bias issues cannot be overlooked. Health datasets used to train AI models may contain biases (e.g., lack of diverse sampling, missing values, and imputation methods), potentially amplifying these biases and leading to unfavorable or inaccurate decisions for specific populations characterized by age, gender, race, geography, or economic status (209, 210). Second, data privacy and ethical concerns are prominent, as sensitive patient information (e.g., genomic data, medical history, behavioral and social data) are prone to leakage, and obtaining informed consent from patients is a major obstacle (211, 212). Lastly, precision medicine faces high costs and resource constraints, with primary care hospitals often lacking the capacity to acquire necessary equipment and technologies, while personalized nursing increases demands on medical resources (207, 213). The widespread implementation of precision medicine is currently constrained by technical complexity, multifactorial determinants, and data-sharing limitations. Addressing these barriers is essential to delivering truly precise and effective personalized healthcare at scale.

To address these challenges, joint efforts are required from governments, healthcare institutions, and research organizations (214). The implementation of artificial intelligence in cardiovascular care must strictly comply with evolving regulatory frameworks designed to ensure patient safety and data privacy. For instance, the European Union's AI Act classifies healthcare-related AI systems as “high-risk”, requiring rigorous conformity assessments and continuous human oversight. Similarly, strict adherence to data protection regulations—such as the General Data Protection Regulation in Europe and the Health Insurance Portability and Accountability Act in the U.S.—is essential to safeguard patient trust and ensure data sovereignty. Healthcare institutions should train their medical staff, equip them with relevant knowledge and skills to improve the quality of personalized nursing. Research institutions should aim to develop new technologies to enhance the technical feasibility of precision medicine. For example, developing more cost-effective gene detection technologies, simplifying complex data analysis algorithms, and lowering the prices of tests and products.

In summary, the continuous development of personalized and precision medicine technologies is expected to improve the cardiovascular health of populations. By integrating multi-omics analysis, machine learning, big data analytics, and remote monitoring technologies, caregivers can provide more precise, personalized, and continuous care services, significantly improving the management of CVDs and patients' quality of life. Current evidence suggests that CVD management, encompassing prevention, diagnosis, treatment, and rehabilitation, will achieve significantly enhanced precision, efficacy, and accessibility in the coming years. Simultaneously, interdisciplinary collaboration is advocated to further drive innovation and development in the field of cardiovascular health, contributing significantly to human health.

## 7 Limitations

This review demonstrates that the integration of precision medicine and personalized nursing into the management of cardiovascular diseases faces multiple challenges, including difficulties in data integration due to the heterogeneity of multi-source data, limitations in the generalizability and potential algorithmic bias of AI models, and ethical and privacy concerns associated with the use of sensitive patient information. Furthermore, there are barriers to clinical implementation—such as high costs, limited technical acceptance, and insufficient model interpretability. Moreover, robust evidence regarding long-term efficacy and cost-effectiveness, gaps in interdisciplinary collaboration, and challenges related to patient adherence and health equity collectively constrain the real-world applicability of this approach. In future, standardized data protocols, explainable AI methodologies, large-scale clinical validation studies, and the establishment of multi-level collaborative networks are needed to promote the translation of this paradigm from theory into clinical practice.

## Glossary

CVDs: cardiovascular diseases
AI: artificial intelligence
CAD: coronary artery disease
WHO: World Health Organization
SEM: scanning electron microscopy
GWAS: genome-wide association study
PRS: polygenic risk score
HF: heart failure
HFPEF: heart failure with preserved ejection fraction
ECG: electrocardiogram
P4 medicine: predictive, preventive, personalized, and participatory medicine
ACNAP: Association of Cardiovascular Nursing & Allied Professions
CHF: chronic heart failure
EHR: electronic health records
PAH: pulmonary arterial hypertension
DCM: dilated cardiomyopathy
WGS: whole-genome sequencing
SNPs: single-nucleotide polymorphisms
scRNA-seq: single-cell RNA sequencing
HCM: hypertrophic cardiomyopathy
snRNA-seq: single-nucleus RNA sequencing
ACS: acute coronary syndrome
T2DM: type 2 diabetes mellitus
NLR: neutrophil-to-lymphocyte ratio
LVDD: left ventricular diastolic dysfunction
MI: myocardial infarction
MR: Mendelian randomization
DIA: data-independent acquisition
AMI: acute myocardial infarction
MVA: malignant ventricular arrhythmia
ASCVD: atherosclerotic cardiovascular disease
PPI: protein-protein interaction
KEGG: Kyoto encyclopedia of genes and genomes
GO: gene ontology
ML: machine learning
CNN: convolutional neural network
RNN: recurrent neural network
LVH: left ventricular hypertrophy
SEN: sensitivity
SPE: specificity
AI-QCT: artificial intelligence-based quantitative coronary tomography
CCTA: coronary CT angiography
QCA: quantitative coronary angiography
LLM: large language model
SVM: support vector machine
OPA: occupational physical activity
SES: socioeconomic status
PM2.5: particulate matter 2.5 micrometers or less in diameter
R/S: religious beliefs and spirituality
NYHA: New York Heart Association
NO: nitric oxide
PTSD: post-traumatic stress disorder
DOH: social determinants of health
CAC: coronary artery calcium.
